# Narciclasine, an isocarbostyril alkaloid, has preferential activity against primary effusion lymphoma

**DOI:** 10.1038/s41598-020-62690-9

**Published:** 2020-03-31

**Authors:** Ramakrishnan Gopalakrishnan, Hittu Matta, Sunju Choi, Preet M. Chaudhary

**Affiliations:** Jane Anne Nohl Division of Hematology and Center for the Study of Blood Diseases, University of Southern California, Keck School of Medicine, Los Angeles, California United States of America

**Keywords:** Drug development, Non-hodgkin lymphoma

## Abstract

Primary effusion lymphoma (PEL) is a subtype of non-Hodgkin lymphoma associated with infection by Kaposi sarcoma-associated herpes virus (KSHV). PEL is an aggressive disease with extremely poor prognosis when treated with conventional chemotherapy. Narciclasine, a natural product present in *Amaryllidaceae* family of flowering plants including daffodils, belongs to a class of molecules termed ‘isocarbostyril alkaloid’. We have found that narciclasine displays preferential cytotoxicity towards PEL at low nanomolar concentrations and is approximately 10 and 100-fold more potent than its structural analogs lycoricidine and lycorine, respectively. Narciclasine arrested cell-cycle progression at the G_1_ phase and induced apoptosis in PEL, which is accompanied by activation of caspase-3/7, cleavage of PARP and increase in the surface expression of Annexin-V. Although narciclasine treatment resulted in a marked decrease in the expression of MYC and its direct target genes,time-course experiments revealed that MYC is not a direct target of narciclasine. Narciclasine treatment neither induces the expression of KSHV-RTA/ORF50 nor the production of infectious KSHV virions in PEL. Finally, narciclasine provides dramatic survival advantages to mice in two distinct mouse xenograft models of PEL. In conclusion, our results suggest that narciclasine could be a promising agent for the treatment of PEL.

## Introduction

Primary effusion lymphoma (PEL) is an aggressive type of non-Hodgkin lymphoma with extremely poor prognosis when treated with conventional chemotherapy. The presence of KSHV in all tumor cells is the defining feature of PEL^1^. Upon diagnosis with PEL, the median survival time of patients is only 3 to 6 months^2^. Therefore, there is an immediate need to identify novel treatment options for PEL.

Narciclasine (also known as lycoricidinol) is a natural product found in daffodils and other flowering plants belonging to the *Amaryllidaceae* (amaryllis) family. Narciclasine has been shown to possess potent anticancer activity against tumors of brain, skin and breast^3^. Earlier studies have shown that translation elongation factor eEF1A is the direct target of narciclasine^4,5^. Further, it has been found that narciclasine triggers actin stress fiber formation by activation of a small GTPase, RhoA^5,6^. Recently, narciclasine was named ‘Molecule of the Week’ by American Chemical Society (ACS) for its potential as a cancer drug.

MYC regulates numerous cellular activities, including signal transduction, cell cycle, proliferation, differentiation and apoptosis. MYC is deregulated in many cancers, and has been implicated in almost a third of all cancers^7^. Even though, the Myc genomic locus is structurally intact in PEL, they modestly overexpress MYC and we have shown that compounds that down regulate MYC expression are effective and selective against PEL^8^.

In this study, we tested the effect of narciclasine and its structural analogs on a panel of cell lines comprising five hematological malignancies. We show that while all the cancer cell lines in our panel were susceptible to narciclasine and its structural analogs, the PEL derived cell lines displayed preferential sensitivity. We further show that preferential activity of narciclasine against PEL is associated with its ability to downregulate MYC.

## Results

### Narciclasine and its structural analogs display preferential cytotoxicity towards PEL

**Figure 1:**
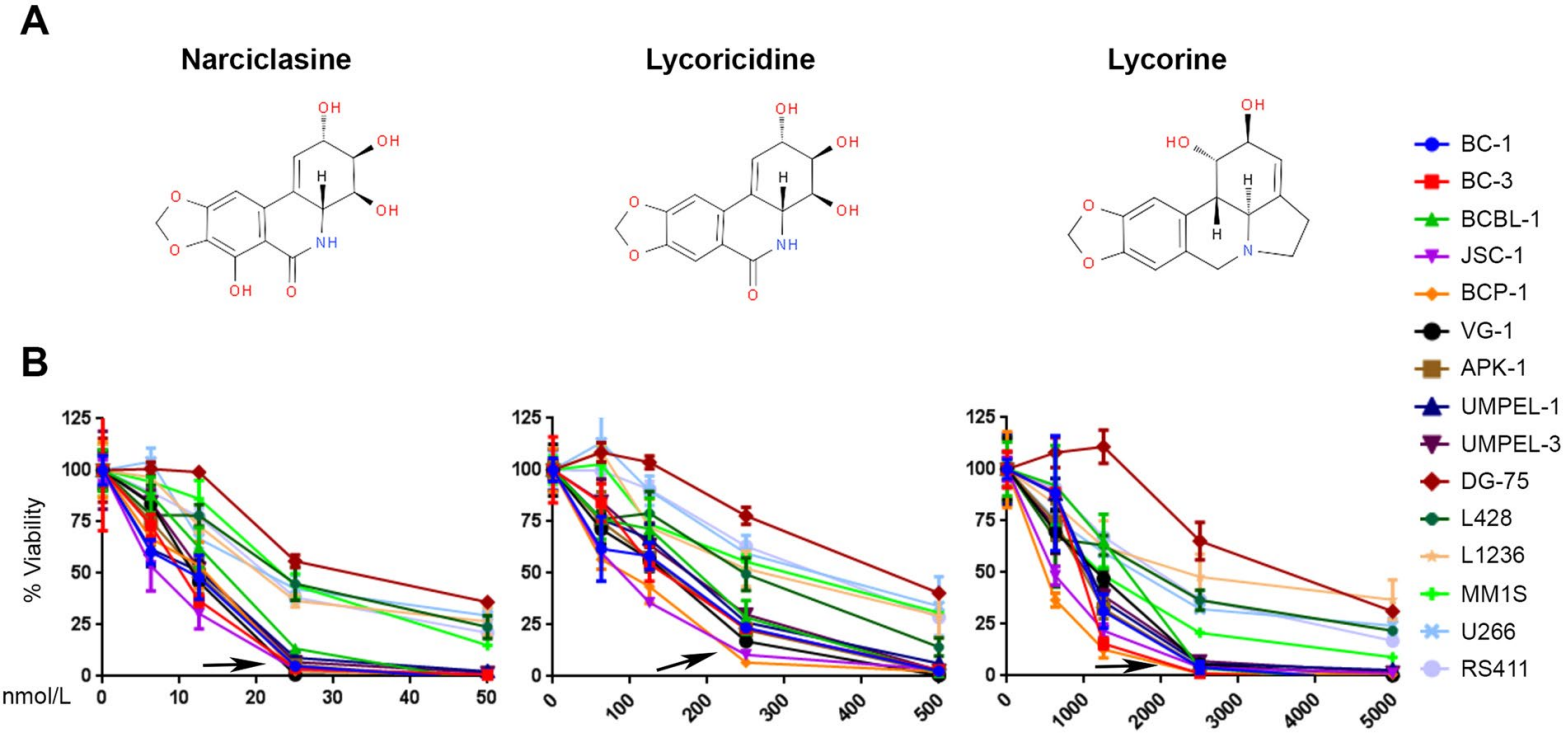
Narciclasine and its structural analogs have preferential cytotoxicity towards PEL. (**A)** Chemical structures of narciclasine, lycoricidine, and lycorine. (**B)** Indicated panel of cell lines were treated with increasing concentrations of narciclasine, lycoricidine, and lycorine for 72 hours. Cell viability was measured using an MTS (3-(4,5-dimethylthiazol-2-yl)-2,5-diphenyltetrazolium bromide) assay. An arrow represents cell lines with preferential sensitivity to the compounds. The values shown are mean ± SE. (*n* = 3) of a representative experiment performed in triplicate for three times.

To determine the effect of narciclasine against PEL, 15 logarithmically growing hematological cancer cell lines representing 5 different cancers were treated with increasing concentrations of narciclasine for 72 hours. Narciclasine displayed preferential cytotoxicity towards PEL cell lines with IC50 ranging from 7 to 14 nM (Fig. 1B & Table 1). In contrast, IC50 of narciclasine for non-PEL cell lines ranged from 22 to 34 nM (Fig. 1B & Table 1). Lycoricidine and lycorine are structural analogs of narciclasine. To identify whether the structural analogs of narciclasine also display preferential cytotoxicity towards PEL, we treated the same panel of hematological cancer cell lines with increasing concentrations lycoricidine and lycorine for 72 hours. Similar to narciclasine, its closely related structural analog lycoricidine also displayed preferential cytotoxicity towards PEL cell lines with IC_50_ ranging from 82 to 162 nM (Fig. 1B & Table 1). In contrast, the IC50 of lycoricidine for non-PEL cell lines ranged from 224 to 426 nM (Fig. 1B & Table 1). Lycorine, the other structural analog of Narciclasine, also displayed a similar trend in cytotoxicity (Fig. 1B & Table 1) although it was much less potent. Thus, even though narciclasine and its structural analogs show similar trend in preferential cytotoxicity towards PEL, the IC50 dose of narciclasine is approximately 10 and 100- fold lower than that of lycoricidine and lycorine, respectively.

**Table 1:** List of cell lines, diseases, and IC_50_ doses of narciclasine and its structural analogs for 72 hours.

Cell line	Disease	IC_50_ (nM)		
		Narciclasine	Lycoricidine	Lycorine
BC-1	PEL	9	115	1030
BC-3	PEL	10	137	916
BCBL-1	PEL	14	162	1405
JSC-1	PEL	7	82	613
BCP-1	PEL	11	83	487
VG-1	PEL	11	122	1029
APK-1	PEL	11	131	937
UMPEL-1	PEL	10	152	1072
UMPEL-3	PEL	12	159	974
DG-75	Burkitt’s Lymphoma	34	426	3549
L428	Hodgkin’s Lymphoma	23	224	1570
L1236	Hodgkin’s Lymphoma	22	274	2501
MM1S	Multiple Myeloma	24	290	1144
U266	Multiple Myeloma	23	336	1615
RS4;11	B-ALL	22	326	1914

Abbreviations - PEL: Primary effusion lymphoma; B-ALL: B cell acute lymphoblastic leukemia; nM: Nanomolar.

### Narciclasine induces cell-cycle arrest in PEL

**Figure 2:**
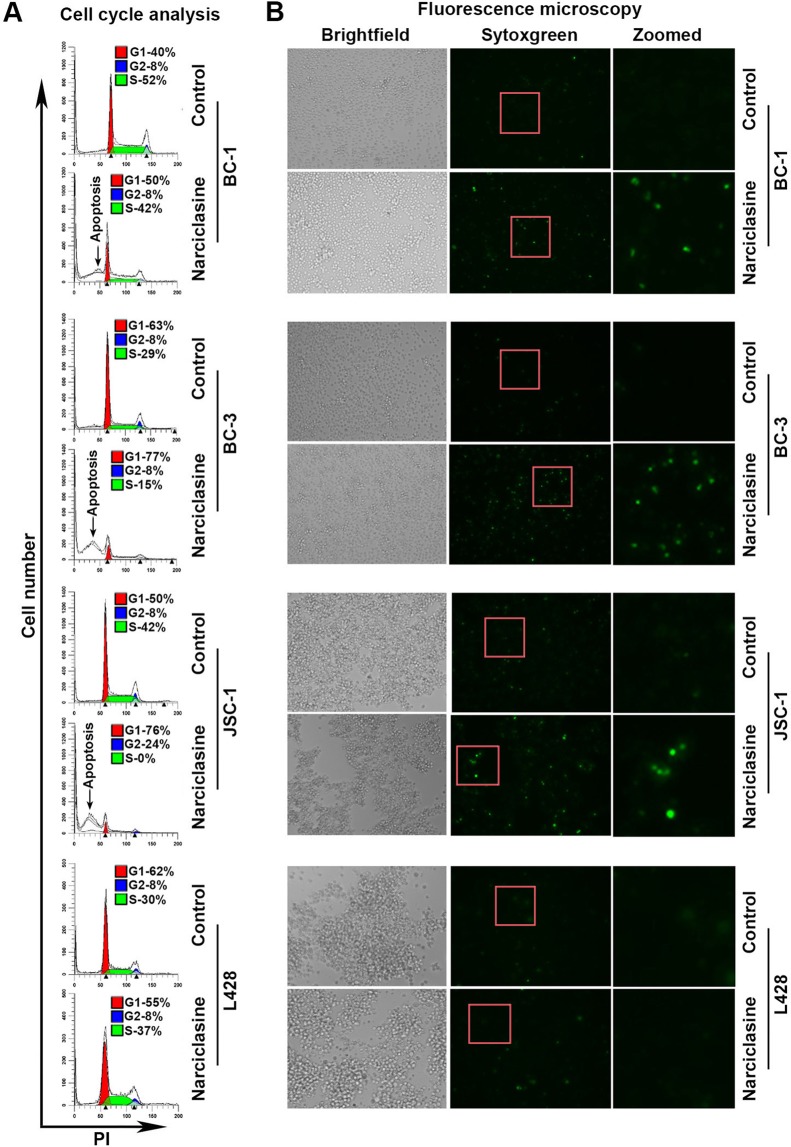
Narciclasine arrested cell-cycle progression. (**A)** Indicated cell lines were treated with narciclasine (25 nM for 36 hours) or DMSO control, fixed with 70% alcohol followed by staining with Propidium Iodide (PI) and analyzed by flow cytometry. (**B)** Indicated cell lines were treated with narciclasine or DMSO control were stained with SYTOX Green, a cell-impermeable nuclear dye that stains the nuclei of dead cells, and were examined under a fluorescence microscope and photographed.

To identify the mechanism by which narciclasine inhibited the growth of PEL cells, we examined the effect of narciclasine on cell-cycle progression using flow cytometry. Treatment of PEL cells with narciclasine resulted in G_1_ arrest as observed by a marked increase in the number of cells in the G_1_ phase along with a concomitant decrease in cells in the S-phase (Fig. 2A). Narciclasine treatment also resulted in a significant increase in the number of cells in sub-G_1_ phase indicative of apoptosis. Further, Narciclasine treated PEL cells stained brightly with SYTOX Green, a membrane-impermeable nuclear dye, indicating loss of membrane permeability accompanying apoptosis (Fig. 2B). In contrast, narciclasine had no major effect on both cell-cycle progression and SYTOX Green staining in the non-PEL derived L428 cells (Fig. 2A,B).

### Narciclasine and its structural analogs induce apoptosis in PEL

**Figure 3:**
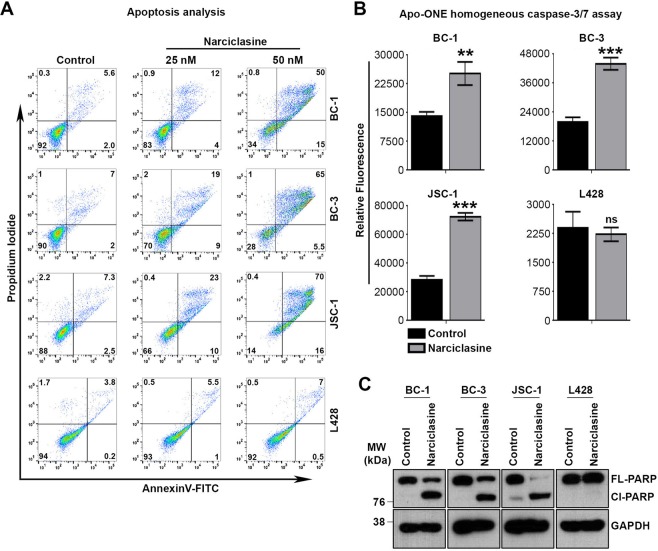
Narciclasine induces apoptosis in PEL cells. (**A)** Indicated cell lines were treated with narciclasine (25 nM for 36 hours) or DMSO control, stained with Annexin V-FITC/propidium iodide, and analyzed for apoptosis by flow cytometry. Data are representative of 3 independent experiments. The gating of cells for analysis were presented in Supplementary Fig. S3. (**B)** Indicated cell lines were treated with narciclasine (25 nM for 36 hours) or DMSO control, followed by measurement of active caspase-3/7 using Apo-ONE homogeneous assay kit. Data are representative of 2 independent experiments. Statistically significant differences were shown by asterisks (**) at a level of p ≤ 0.01, and (***) at a level of p ≤ 0.001. ns – not significant. (**C)** BC-1, BC-3, JSC-1 and L428 cell lines were treated with narciclasine (25 nM for 48 hours) or DMSO control, followed by western blotting of whole cell lysates for cleavage of PARP and GAPDH (loading control). Cl – Cleaved; FL – Full Length. Samples were derived from the same experiments, loading controls were from the same blot and the blots were processed in parallel. Original raw blots are presented in Supplementary Fig. S4. Blots are representative of 3 independent experiments.

To confirm whether narciclasine induces apoptotic cell death in PEL, we treated the PEL cell lines BC-1, BC-3 and JSC-1 with narciclasine for 36 hours followed by staining with AnnnexinV-FITC. Moderate and dramatic increases in annexinV-FITC staining were observed in PEL cells treated with narciclasine at the doses of 25 nM (2 to 3-fold) and 50 nM (8 to 10-fold), as compared with cells that were treated with a vehicle control (Fig. 3A). In contrast, only a mild increase in annexinV-FITC staining was observed in a non-PEL cell line (L428) that had been treated with narciclasine under similar conditions (maximum of 2-fold at 50 nM), confirming the preferential activity of narciclasine towards PEL (Fig. 3A). Consistent with the above results, narciclasine treatment activated caspase-3/7 and increased PARP cleavage, two hallmarks of apoptosis, only in PEL cell lines (Fig. 3B,C). Similar results were obtained with lycoricidine and lycorine (Supplementary Fig. S1).

### MYC is not a direct target of narciclasine

**Figure 4:**
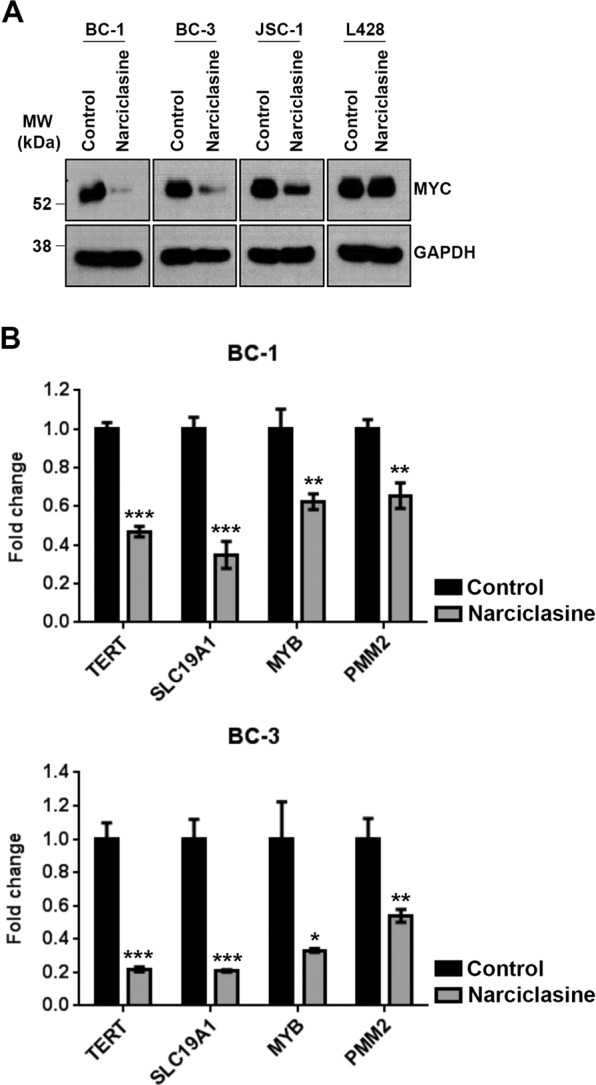
Narciclasine downregulates MYC. (**A)** BC-1, BC-3, JSC-1 and L428 cell lines were treated with narciclasine (25 nM for 48 hours) or DMSO control, followed by western blotting of whole cell lysates for MYC and GAPDH (loading control). Samples were derived from the same experiments, loading controls were from the same blot and the blots were processed in parallel. Original raw blots are presented in Supplementary Fig. S5. Blots are representative of at least 3 independent experiments. (**B)** BC-1 and BC-3 cell lines were treated with narciclasine (50 nM for 24 hours) or DMSO control followed by qRT-PCR analysis of *TERT, SLC19A1, MYB* and *PMM2* mRNA (direct target genes of MYC protein). Real-time PCR reactions were carried out in triplicate and the data were presented as fold change in target gene expression (mean ± SE) from a representative of 2 independent experiments. Statistically significant differences were shown by asterisks (*) at a level of p ≤ 0.05, (**) at a level of p ≤ 0.01, and (***) at a level of p ≤ 0.001.

**Figure 5:**
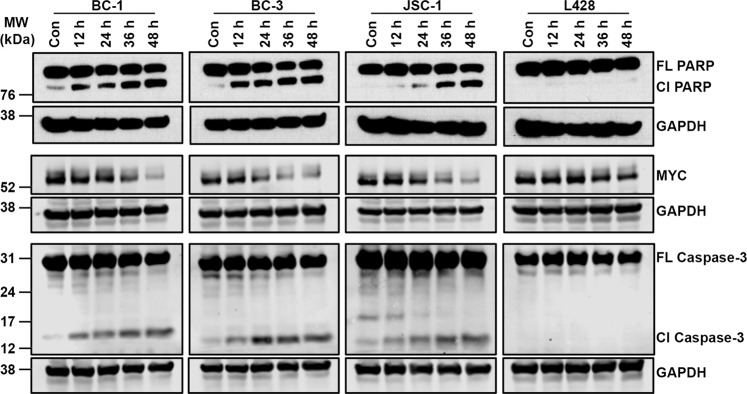
MYC is not a primary target of narciclasine. BC-1, BC-3, JSC-1 and L428 cell lines were treated with narciclasine (25 nM for 12, 24, 36 and 48 hours) or DMSO control, followed by western blotting of whole cell lysates for PARP, MYC, Caspase-3 and GAPDH (loading control). Cl – Cleaved; FL – Full Length. Samples were derived from the same experiments, loading controls were from the same blot and the blots were processed in parallel. Original raw blots are presented in Supplementary Figs. S6–S8.

Recently, we have shown that the agents that downregulate MYC are selectively toxic to PEL^8,9^. Since, narciclasine also display preferential activity against PEL, we tested the effect of narciclasine on the expression of MYC in PEL. BC-1, BC-3 and JSC-1 cells were treated with narciclasine (25 nM) for 48 hours followed by immunoblotting for MYC and GAPDH (loading control). As shown in Fig. 4A, narciclasine treatment resulted in a dramatic decrease in the level of MYC in PEL cell lines. In contrast, narciclasine had no significant inhibitory effect on MYC expression in L428, a non-PEL cell line (Fig. 4A). We used qRT–PCR analysis to examine the effect of narciclasine on the expression of four known MYC target genes *TERT, SLC19A1, MYB* and *PMM2*. Narciclasine treatment induced a significant reduction in expression of all four MYC target genes in both BC-1 and BC-3 cell lines (Fig. 4B). The above results indicated that expression of MYC is downregulated by narciclasine in PEL. To check, whether MYC is the primary target of narciclasine, we performed time-course experiments by treating PEL cells with narciclasine for 12, 24, 36 and 48 hours followed by immunoblotting for MYC, PARP and caspase-3. As shown in Fig. 5, treatment of PEL cells with narciclasine for 12 hours resulted in cleavage of PARP and caspase-3 cleavage, indicating induction of apoptosis. However, a significant reduction in the level of MYC was seen only after 36 hours of treatment (Fig. 5). Collectively, the above results suggest that although MYC expression is downregulated in PEL upon treatment with narciclasine, it is not a direct target of narciclasine.

### Inhibiting Rho pahway has no effect on the activity of narciclasine in PEL

**Figure 6:**
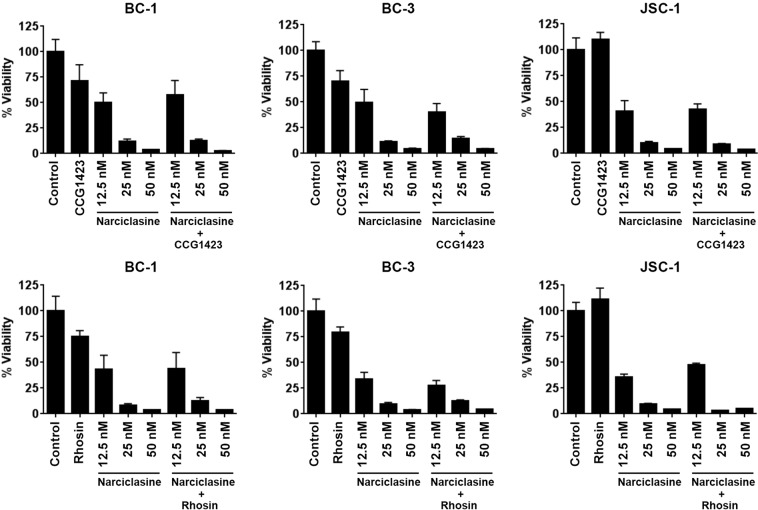
Rho inhibitors have no effect on the activity of narciclasine in PEL. Indicated cell lines were treated with 10 μM of Rho pathway inhibitors CCG1423 and Rhosin.HCl in the presence or absence of indicated concentration of narciclasine for 72 hours. Cell viability was measured using an MTS (3-(4,5-dimethylthiazol-2-yl)-2,5-diphenyltetrazolium bromide) assay. The values shown are mean ± SE. (*n* = 3) of a representative experiment performed in triplicate.

Narciclasine has been shown to induce stress fiber formation by activating RhoA in gliobastoma cells^6^. However, in PEL cells (BC-1 and BC-3) narciclasine did not induce stress fiber formation as observed by F-actin staining (data not shown). Further, two known inhibitors of Rho pathway, CCG1423 and Rhosin-HCl^10,11^, did not have any effect on the activity of narciclasine in PEL cells (Fig. 6). These results are not surprising because activation of Rho and stress fiber formations are usually observed in adherent cells^12^. Collectively, these results indicate that Rho pathway has no role in the activity of narciclasine in PEL.

### Lack of induction of lytic replication by Narciclasine in PEL

**Figure 7:**
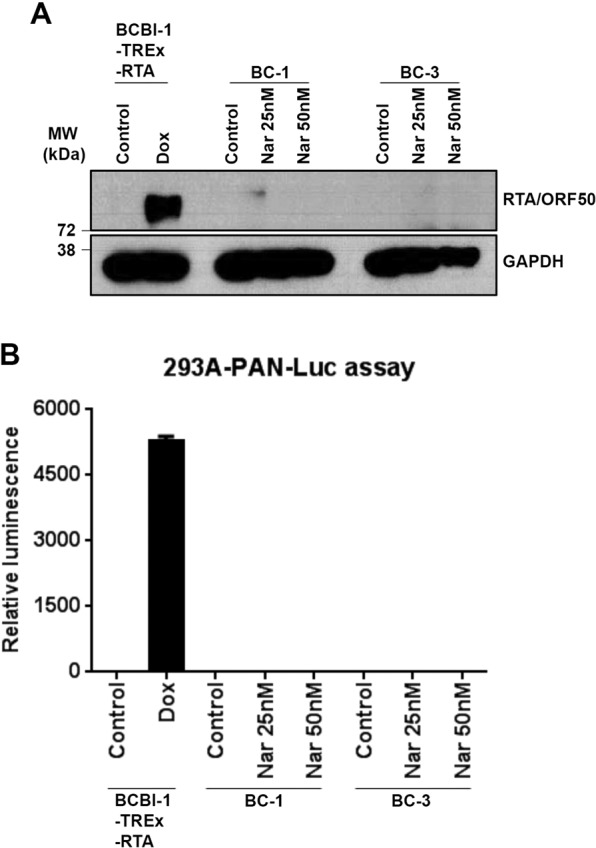
Narciclasine treatment does not induce lytic reactivation of KSHV. (**A)** Western blotting analysis of KSHV RTA/ORF50 and GAPDH (loading control) in indicated PEL cell lines treated with narciclasine for 48 hours. Expression of RTA in BCBL-TREx-RTA cells treated doxycycline (500 ng/ml for 48 hours) serves as a positive control. Samples were derived from the same experiments, loading controls were from the same blot and the blots were processed in parallel. Original raw blots are presented in Supplementary Fig. S9. Blots are representative of at least 2 independent experiments. (**B)** Cell free supernatants collected from narciclasine treated PEL cells were used to infect 293A-PAN-Luc cells, which expresses the firefly luciferase gene under the control of KSHV PAN promoter and responds to infection with KSHV. Cell free supernatant from doxycycline treated BCBL-TREx-RTA cells were used as positive control. Data are representative of 2 independent experiments performed in duplicate.

The defining feature of PEL is the presence of KSHV in all tumor cells. Disruption of Myc by shRNA results in the induction of KSHV replication and transcription activator (RTA), a master regulator and marker for lytic reactivation^13^. Therefore, a potential concern is that narciclasine treatment may induce RTA expression and lytic reactivation of KSHV. Interestingly, treatment of PEL cells with narciclasine did not induce RTA expression as determined by immunoblotting (Fig. 7A). As a positive control, we used BCBL-TREx-RTA cells, which express RTA from tetracycline-inducible promoter and undergo a complete cycle of viral replication upon treatment with doxycycline (Fig. 7A). Earlier, we have made a reporter cell line termed 293A-PAN-Luc, which expresses the firefly luciferase gene under the control KSVH PAN promoter and responds to infectious KSHV virions by upregulating the luciferase activity^14^. To test whether narciclasine treatment of PEL cells produces infectious KSHV virions, cell-free supernatants from narciclasine-treated PEL cells and doxycycline-treated BCBL-TREx-RTA (positive control) were incubated with 293A-PAN-Luc cells for 48 hours in the presence of polybrene, followed by measurement of firefly luciferase activity. A striking increase in luciferase activity was observed in wells that were incubated with supernatants from doxycycline treated BCBL-TREx-RTA cells (Fig. 7B). In contrast, the luciferase activity of the wells treated with supernatants from narciclasine treated PEL cells stayed near the base line (Fig. 7B). These results suggest that narciclasine treatment neither reactivates KSHV lytic cycle nor produces infectious virions.

### Narciclasine displays potent *in vivo* growth inhibitory activity in orthotopic CLX and PDX mouse models of PEL

**Figure 8:**
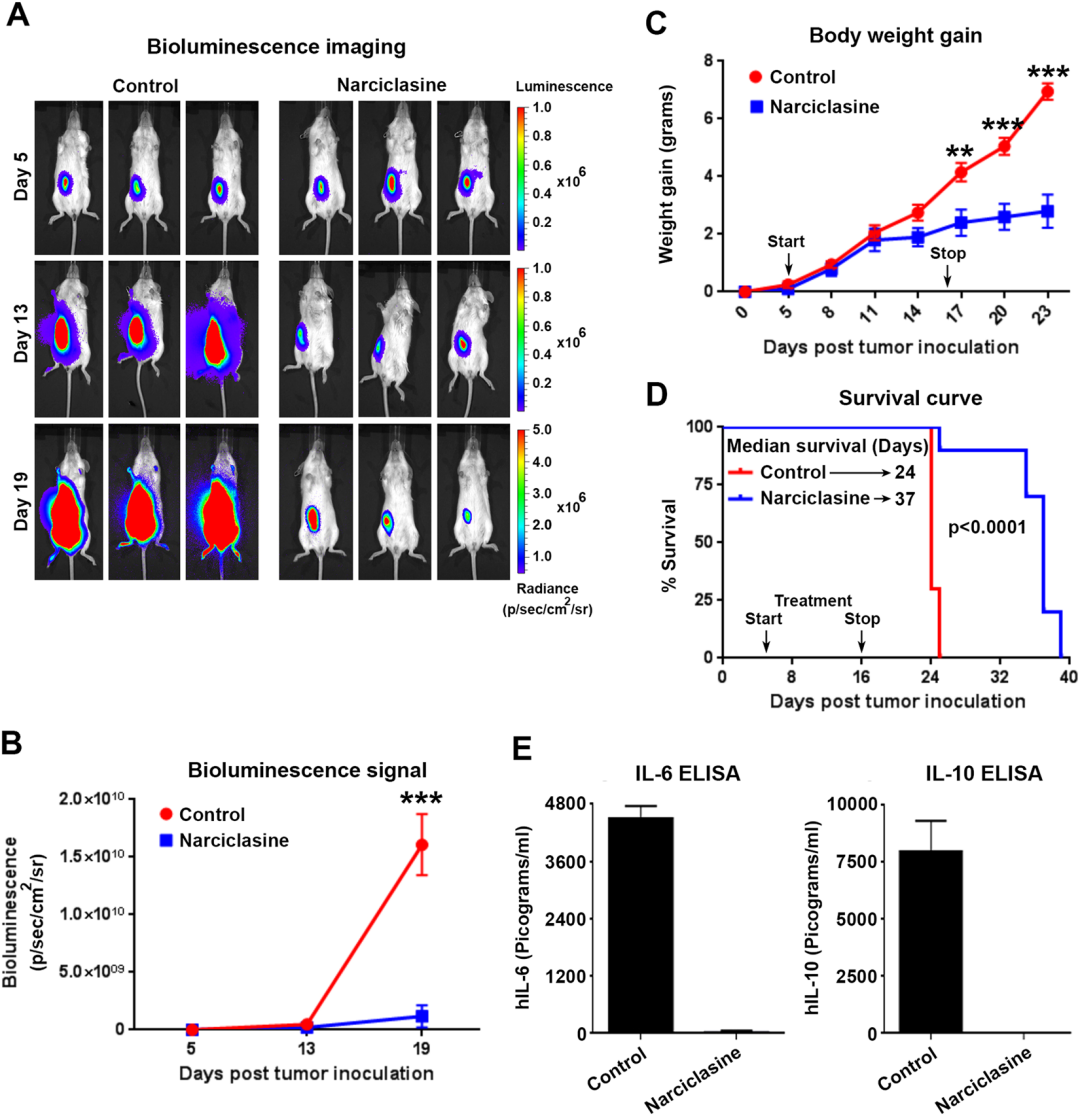
Narciclasine inhibits growth of PEL in an orthotopic cell line xenograft (CLX) model. (**A)** 6-week old female NOD-Scid-Gamma (NSG) mice were injected intraperitoneally with 1 × 10^7^ BC-1 cells stably expressing firefly luciferase gene (BC-1-Fluc). 5 days after tumor inoculation Bio-luminescence Imaging (BLI) was performed to confirm the presence of tumors in mice. Post confirmation, mice were randomly divided into vehicle control or narciclasine (1 mg/kg b.w. intraperitoneally daily for 12 days) groups. Representative serial BLI images of mice on days 5, 13 and 19 of indicated treatment groups are shown. (**B)** Tumor burden as measured by relative luminescence measurements of serial BLI imaging from vehicle control and narciclasine treated mice are shown. (**C)** Body weight gain of mice injected with BC-1-Fluc cells followed by indicated treatments over the period of experiment. Black arrows indicate start (day 5) and stop (day 16) of narciclasine treatment. Asterisks indicate significance (**) at a level of p ≤ 0.01, and (***) at a level of p ≤ 0.001. (**D)** Survival curves (Kaplan-Meier) of mice bearing orthotopic BC-1-Fluc cells treated with vehicle control and narciclasine (n = 10 in each group). The survival curve was generated in GraphPad Prism 5 software and statistical values for the curves are calculated by log rank (Mantel–Cox) test. (**E**) Levels of hIL-6 and hIL-10 in plasma of animals on day19 after tumor inoculation in vehicle control or narciclasine treatment groups.

To check the *in vivo* efficacy of narciclasine against PEL, we employed two distinct mouse models of PEL – a Cell Line Xenograft (CLX) model using BC-1 cells expressing an RSV-promoter-driven firefly luciferase (BC-1-Fluc) and a Patient Derived Xenograft (PDX) model using UMPEL-1 cells^15^. In the CLX model, 1 × 10^7^ BC-1-Fluc cells were intraperitoneally injected into NSG mice on day 1. Bioluminescence imaging (BLI) was performed on day 5 to confirm the presence of tumors (Fig. 8A). Animals with established tumors were randomly assigned to vehicle control (10% HPCD) or narciclasine (1 mg/kg once daily for 12 days). Narciclasine treatment inhibited the growth of established PEL as seen by significant decreases in the BLI signal (Fig. 8A,B) and body weight gain, a surrogate for tumor-induced ascites (Fig. 8C). Further, there was a striking increase in the median survival of mice that received narciclasine (median survival = 37 days) in comparison to vehicle control mice (median survival = 24 days) (Fig. 8D). BC-1 cells produce and secrete cytokines IL-6 and IL-10. As another surrogate marker for the presence of tumor cells, we measured the levels of circulating human IL-6 and IL-10 in the NSG mice bearing BC-1-Fluc cells on day 19 after tumor inoculation. As shown in Fig. 8E, circulating human IL-6 and IL-10 were readily detected in the plasma of NSG mice bearing BC-1-Fluc cells that were treated with vehicle control. In contrast, no circulating IL-6 and IL-10 were seen in mice treated with narciclasine, confirming the effective control of PEL by narciclasine *in vivo*.

**Figure 9:**
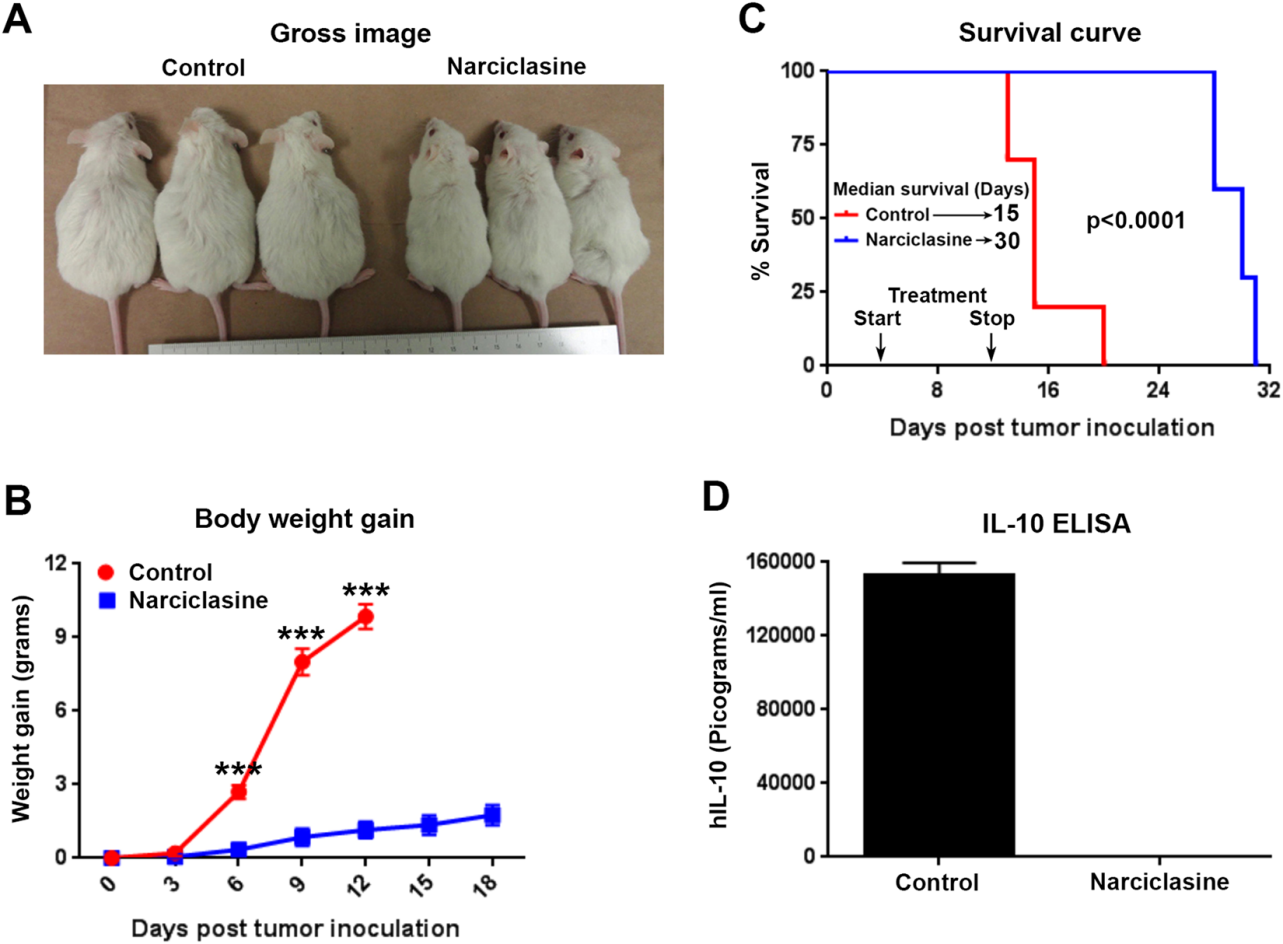
Narciclasine exhibits potent activity in a Patient-Derived Xenograft (PDX) model of PEL. (**A)** 6-week old female NSG mice were injected intraperitoneally with 1 × 10^6^ UMPEL-1 cells. 4 days after tumor inoculation, mice were randomly divided into vehicle control or narciclasine (1 mg/kg b.w. intraperitoneally daily for 9 days) treatment groups. Gross representative images of mice on day 12 after tumor inoculation of indicated treatment groups were shown. (**B)** Body weight gain of mice injected with UMPEL-1 cells followed by treatment with vehicle control and narciclasine over the period of experiment. Statistically significant differences are shown by asterisks (***) at a level of p ≤ 0.001. (**C)** Survival curves (Kaplan-Meier) of mice bearing orthotopic UMPEL-1 cells treated with vehicle control and narciclasine (n = 10 in each group). The survival curve was generated in GraphPad Prism 5 software and statistical values for the curves are calculated by log rank (Mantel–Cox) test. Black arrows indicate start (day 4) and stop (day 12) of narciclasine treatment. (**D)** Circulating level of hIL-10 on day 12 after tumor inoculation in vehicle control or narciclasine treatment groups.

For PDX model, 1 × 10^7^ patient-derived UMPEL-1 cells were intraperitoneally injected in to NSG mice. 4 days after tumor inoculation, animals were randomly assigned to vehicle control and narciclasine (1 mg/kg once daily for 9 days). As shown in Fig. 9A,B, treatment with narciclasine inhibited the growth of patient-derived UMPEL-1 cells as seen by a significant decrease in body weight gain over the experimental period. Moreover, there was a 2-fold increase in the median survival of mice that received narciclasine (median survival = 30 days) as compared with vehicle control mice (median survival = 15 days) (Fig. 9C). In contrast to BC-1 cells, UMPEL-1 cells produce and secrete only human IL-10. We did not detect any circulating human IL-10 in animals inoculated with patient-derived UMPEL-1 cells that were treated with narciclasine (Fig. 9D). In contrast, significant amount of human IL-10 was detected in vehicle control treated animals bearing patient-derived UMPEL-1 cells. In the same setting, a group of animals were also treated with narciclasine analog – lycoricidine (1 mg/kg once daily for 9 days). As shown in Supplementary Fig. S2, lycoricidine has a moderate but significant inhibitory effect on the growth of patient-derived UMPEL-1 cells as determined by a significant decrease in body weight gain, increase in median survival (by 6 days) along with a significant decrease in circulating human IL-10. The moderate effect of lycoricidine *in vivo* is in clear contrast to the striking effect of narciclasine at similar treatment doses, which confirms the *in vitro* cytotoxicity data suggesting the superior activity narciclasine over its structural analogs.

## Discussion

PEL accounts for approximately 4% of all HIV-related non-Hodgkin lymphoma and is universally associated with KSHV infection^16^. The median overall survival of PEL is only 4.8 months, indicating an urgent need to identify new therapeutic options for this disease^17^. In this study, we found that narciclasine and its structural analogs have preferential activity against PEL. Preclinical studies of narciclasine in other cancers have shown that its mechanism of action involves inhibition of translation elongation factor eEF1A^4^, activation of RhoA^6^, and induction of autophagy^18^. It is possible that all these pathways play a role in the activity of the narciclasine against PEL. However, all those pathways are essential for the survival of both cancer cells and normal cells. In this study, we found that all the cancer cells in the panel were susceptible to narciclasine induced cell death at higher doses, while cell lines representing PEL succumbed to narciclasine at doses that were approximately 2.5-fold lower than the cell lines representing other cancers. This led us to hypothesize that narciclasine may downregulate MYC in PEL as MYC is essential for the survival of PEL and molecules that target MYC are effective and selective against PEL^8,9^. Indeed, we found that narciclasine downregulated the expression of MYC with a concomitant decrease in the expression of its direct target genes. However, time-course experiments in PEL upon treatment with narciclasine revealed that apoptosis was initiated before the downregulation of MYC, suggesting that MYC is not a direct target of narciclasine in PEL.

Downregulating MYC expression in PEL using shRNA leads to lytic reactivation of KSHV by activating RTA/ORF50, the master regulator of KSHV lytic cycle^13^. As lytic reactivation of KSHV is believed to promote KSHV tumorigenesis, it can be a potential safety issue with the use of narciclasine in PEL^19^. However, we found that narciclasine treatment neither activated RTA nor induced any infectious KSHV particles, thereby allaying those safety concerns. These results are consistent with our observation that MYC is not a direct target of narciclassine.

Narciclasine has been shown to activate Rho signaling and induce stress fiber formation in glioblastoma cells^6^. However, our study suggests that Rho pathway has no role in the activity of narciclasine in PEL. The KSHV genome contains several genes that mimics the activity of cellular genes, thereby activating multiple oncogenic signaling pathways (Akt, MAPK, NF-κB, Notch, Wnt/β-catenin)^20^. At present, it is not known whether narciclasine has any effect on any of the above-mentioned oncogenic signaling pathways present in PEL. Therefore, further studies are needed to identify the primary target of narciclasine in PEL. Apart from its potent anticancer activities, narciclasine has been shown to possess anti-inflammatory properties by inhibiting NF-κB pathway in murine models of inflammation^21^. NF-κB pathway is constitutively active in a number of PEL cell lines^22–24^ and has been shown to be essential for their survival and proliferation^24,25^. Therefore, it is conceivable that inhibition of survival pathways, such as NF-κB, also contributes to the activity of narciclasine against PEL cells.

Even though narciclasine has shown impressive activity in various pre-clinical studies of cancers, it has never been tested in a human clinical trial. A potential bottleneck for the clinical testing of narciclasine is its limited availability. Until now, narciclasine has been isolated from the bulbs of *narcissus*. However, recent studies have successfully demonstrated organic routes for chemical synthesis of narciclasine and its analogs^26,27^. In this study, we show that compounds that are structurally related to narciclasine also display similar activity, albeit at higher doses. Therefore, structural modifications of narciclasine can be made to improve its efficacy and bio-availability. Finally, it is pertinent to note that concentrations as high as ~250 nM of narciclasine were readily achievable in murine pharmacokinetics studies, indicating the presence of a significant therapeutic window, which can be exploited for the treatment of PEL^3^.

## Materials and Methods

### Cell lines

BC-1, BC-3, BCBL-1, JSC-1, BCP-1, VG-1 and APK-1 were obtained from Dr. Jae Jung (University of Southern California, CA, USA). UMPEL-1 and UMPEL-3 were provided by Dr. Izidore Lossos and Dr. Juan Ramos, respectively (both from University of Miami, Miami, FL, USA). DG-75 was obtained from Dr. Alan Epstein (University of Southern California, CA, USA). RS4.11 was purchased from American type culture collection, Manassas, VA, USA. L428 and L1236 cells were obtained from Dr. Markus Mapara (Columbia University Medical Center, NY, USA). MM.1 S was obtained from Dr. Alan Lichenstein (Veterans affairs hospital, Los Angeles, CA, USA) and U266 was provided by Dr. Gregor Adams (University of Southern California, CA, USA). All the cells were grown in conditions as described previously^9,28^. 293 A cells (Invitrogen) were grown in Dulbecco’s modified Eagle’s medium supplemented with 10% (v/v) fetal bovine serum. The cells were maintained at 37 °C and 5% CO_2._ After the cell lines were received they were expanded and multiple vials were frozen in liquid nitrogen. None of the cell lines used in this study were cultured continuously for longer than 3 months. After 3 months, a new frozen vial was thawed and used for experiments. PEL cells were authenticated by their expression of KSHV-LANA. No further authentication of cell lines characteristics was done.

### Reagents

Narciclasine (NSC 266535) and lycoricidine (NSC 349155) were obtained from the Developmental Therapeutics Program (DTP) of National Cancer Institute (NCI). Lycorine was obtained from Enzo Life Sciences (BML-GR313). Phenazine methosulfate (P9625); Dimethyl sulfoxide (D2650); Doxycycline (D9891) and 2-Hydoxypropyl-β-cyclodextrin (HPCD – 332607) were obtained from Sigma-Aldrich. Apo-ONE® Homogeneous Caspase-3/7 assay kit (G7792) and Cell Titer 96® Aqueous MTS reagent powder (G1111) were obtained from Promega. FITC Annexin V apoptosis detection kit was obtained from BD biosciences (556547). Human IL-6 (DY206) and IL-10 (DY217B) were measured using the Duoset ELISA kit from R&D systems. CCG1423 (5233) and Rhosin.HCl (5033) was obtained from Tocris. Phalloidin-iFluor 488 reagent for F-actin staining was obtained from Abcam (ab176753).

### Cell viability, cell-cycle and apoptosis analysis

Cells from exponentially growing cultures were plated in untreated flat-bottom 96 well plates at a density of 1 × 10^4^ cells/well, treated with an increasing concentration of the drugs and subsequently assessed for cell viability using the MTS reagent (3-4,5-dimethylthiazol-2yl)-5-(3-carboxy -methoxyphenyl)-2-(4-sulfophenyl)-2H-tet razolium, inner salt) following the manufacturer’s instructions. Percent cell survival was calculated based on the reading of cells grown in the presence of DMSO control, as described previously^29^. IC_50_ values were calculated using Graphpad prism. Experiments were performed in triplicate. Cell-cycle analysis was done as described previously^30^. Apoptosis was analyzed using Apo-ONE^®^ Homogeneous Caspase-3/7 Assay kit and FITC AnnexinV apoptosis detection kit I as per manufacturer instructions, as described previously^28^.

### Preparation of cell lysates, western blotting and antibodies

Cells were lysed in lysis buffer containing 20 mM sodium phosphate (pH 7.4), 150 mM NaCl, 0.1% Triton X-100, 0.2 M PMSF, and 10% glycerol supplement with a protease inhibitor mixture tablet (Roche). Whole cell extracts were resolved by SDS-polyacrylamide gel electrophoresis, transferred to nitrocellulose membrane, and probed with the indicated primary antibodies, as described previously^30,31^. Primary antibodies used in these experiments were from the following sources: PARP (9542, Cell Signaling); MYC (1472-1, Epitomics); Caspase-3 (9662, Cell Signaling); GAPDH (1107018, Ambion).

### Real-time RT-PCR

PEL cells treated with narciclasine were harvested to extract total RNA using the RNeasy mini kit (Qiagen) and cDNA was synthesized using reverse transcriptase enzyme Superscript II (Invitrogen). Real-time quantitative reverse transcript-polymerase chain reaction (qRT-PCR) was performed with SYBR Green using gene-specific PCR primers listed in Supplementary Table 1. Samples were run in triplicate, and PCR was performed by an ABI Step One Plus thermocycler (Applied Biosystems). GAPDH was used as housekeeping gene and qRT-PCR data (Ct values) was analyzed using the 2^−∆∆ CT^ method as described earlier^14^. The qRT-PCR data was presented as fold change in target gene expression ± standard error.

### Luciferase assay for the presence of KSHV infectious virions

293A-PAN-Luc cells engineered to express a stably integrated copy of PAN promoter-driven luciferase reporter construct were plated in 24 well plates and treated with cell-free supernatants from PEL cell lines treated with narciclasine or doxycycline (500 ng/ml) treated BCBL-TREx-RTA supernatants for 48 hours in the presence of polybrene (8 µg/ml). Post incubation, cells were lysed to assay the firefly luciferase activity as described earlier^32^.

### Cell line xenograft (CLX) mouse model of PEL

A total of 1 × 10^7^ BC-1 cells stably expressing firefly luc were injected intraperitoneally into approximately 6-week-old female NOD.Scid-Gamma (NSG) mice (purchased from Jackson laboratory or from the breeding colony maintained in house). To assess establishment of tumors, mice were imaged 5 days post inoculation using an IVIS Spectrum Imaging system (Perkin Elmer’s, Waltham, MA, USA). Animals were randomly assigned into two groups that were treated with once daily intraperitoneal injections of vehicle control (10% HPCD) or narciclasine (1 mg/kg) for 12 days, continuously. The animals were monitored for survival and body weight gain was measured every 3 days as a surrogate measure of tumor progression^9^. All animal handling procedures were performed with the approval of the University of Southern California (USC) Institutional Animal Care and Use Committee (IACUC), in accordance with ethical guidelines and regulations.

### Patient derived xenograft (PDX) mouse model of PEL

A total of 1 × 10^7^ patient derived UMPEL-1 cells that were passaged only in mice were injected intraperitoneally into approximately 6-week-old female NSG mice^15^. 4 days after tumor cells inoculation, mice were randomly divided into three groups and were treated with a once daily intraperitoneal injections of vehicle control (10% HPCD) or narciclasine (1 mg/kg) or lycoricidine (1 mg/kg) for 9 days. The animals were monitored for survival and body weight gain over the experimental period. All animal handling procedures were performed with the approval of the University of Southern California (USC) Institutional Animal Care and Use Committee (IACUC), in accordance with ethical guidelines and regulations.

### Statistical analysis

Two-tailed unpaired Student *t* test was used to test for differences between 2 groups. Differences with a *P* ≤ 0.05 were considered statistically significant. All experiments were repeated a minimum of two times.

## Supplementary information

Supplementary Information-Narciclasine in PEL.

## Data Availability

The data and reagents will be available up on request to senior author P.M.C. and/or R.G.
