# Supplementary material for: Narciclasine, an isocarbostyril alkaloid, has preferential activity against primary effusion lymphoma
Source: Sci Rep. 2020 Mar 31;10:5712. doi: 10.1038/s41598-020-62690-9 (PMC7109099; doi:10.1038/s41598-020-62690-9)
Supplement: Supplementary file 1 — Supplementary Information-Narciclasine in PEL. [file 41598_2020_62690_MOESM1_ESM.pdf]

Supplementary Information

Narciclasine, an isocarbostyril alkaloid, has preferential activity against primary effusion lymphoma

Ramakrishnan Gopalakrishnan, Hittu Matta, Sunju Choi and Preet M. Chaudhary*
Jane Anne Nohl Division of Hematology and Center for the Study of Blood Diseases, University of Southern California, Keck School of Medicine, Los Angeles, California, United States of America.

***Corresponding author:** Preet M. Chaudhary, M.D., Ph.D.

Email: preet.chaudhary@med.usc.edu

Supplementary figure S1

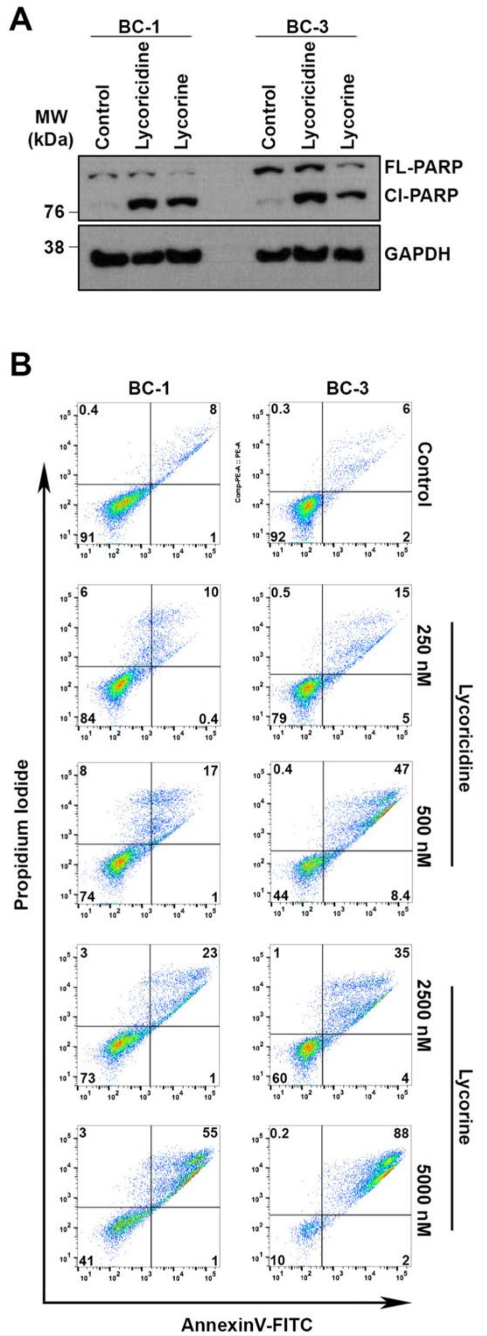

Supplementary figure S1. Structural analogs of narciclasine – lycoricidine and lycorine induces apoptosis in PEL cells. **A)** BC-1 and BC-3 cell lines were treated with lycoricidine (250 nM for 48 hours) or lycorine (2500 nM for 48 hours) or DMSO control, followed by western blotting of whole cell lysates for cleavage of PARP and GAPDH (loading control). Cl – Cleaved; FL – Full Length. Samples were derived from the same experiments, loading controls were from the same blot and that blots were processed in parallel. Original raw blots are presented in Supplementary Figure S6. **B)** Indicated cell lines were treated with lycoricidine (250 nM and 500 nM for 36 hours) or lycorine (2500 nM and 5000 nM for 36 hours) or DMSO control, stained with Annexin V-FITC/propidium iodide, and analyzed for apoptosis by flow cytometry.

Supplementary figure S2

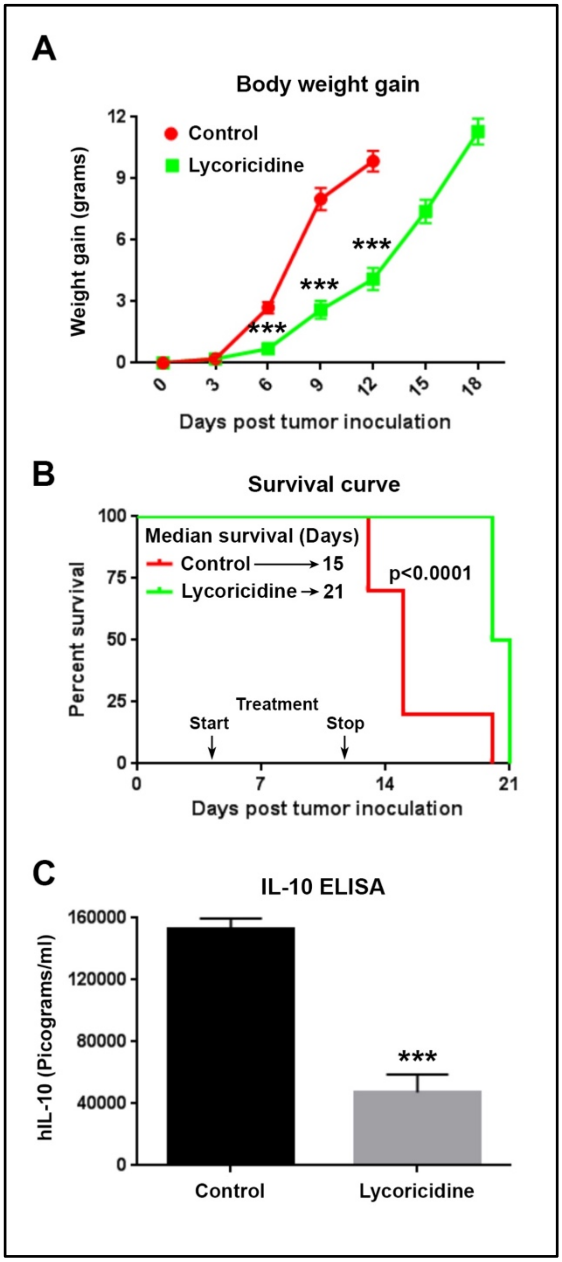

Supplementary figure S2. Lycoricidine (narciclasine analog) exhibited moderate but significant activity in a Patient-Derived Xenograft (PDX) model of PEL. **A)** Body weight gain of mice injected with UMPEL-1 cells followed by treatment with vehicle control and lycoricidine over the period of experiment. **B)** Survival curves (Kaplan-Meier) of mice bearing orthotopic UMPEL-1 cells treated with vehicle control and lycoricidine (n=10 in each group). The survival curve was generated in GraphPad Prism 5 software and statistical values for the curves are calculated by log rank (Mantel–Cox) test. Black arrows indicate start (day 4) and stop (day 12) of narciclasine treatment. **C)** Circulating level of hIL-10 on day 12 after tumor inoculation in vehicle control or narciclasine treatment groups. Statistically significant differences are shown by asterisks (***) at a level of $p \leq 0.001$.

Supplementary Table 1. Sequence of primers used for qRT-PCR.

Gene	Forward Primer	Reverse Primer
GAPDH	GAAGGTGAAGGTCGGAGTC	GAAGATGGTGATGGGATTTC
MYB	CTTTCCACAGGATGCAGGTT	GCACCAGCATCAGAAGATGA
TERT	ATCAGCCAGTGCAGGAACTT	AGCTGACGTGGAAGATGAGC
PMM2	GGTTCTGGGGTCTGTGAAGA	GTCTTTCCTGATGGATGGGA
SLC19A1	ATGGCCCCCAAGAAGTAGAT	GTCAACACGTTCTTTGCCAC

Supplementary figure S3: Gating of cells for apoptotis analysis related to figure 3A in manuscript

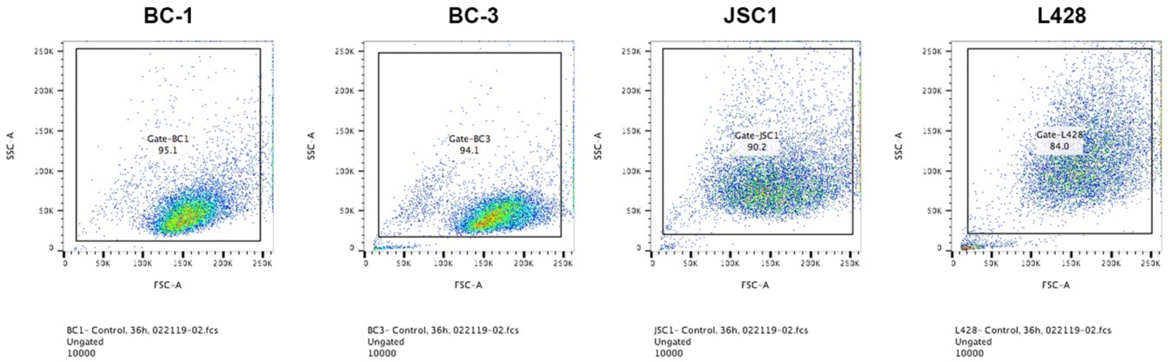

Supplementary figure S4: Full raw blots of Figure 3C

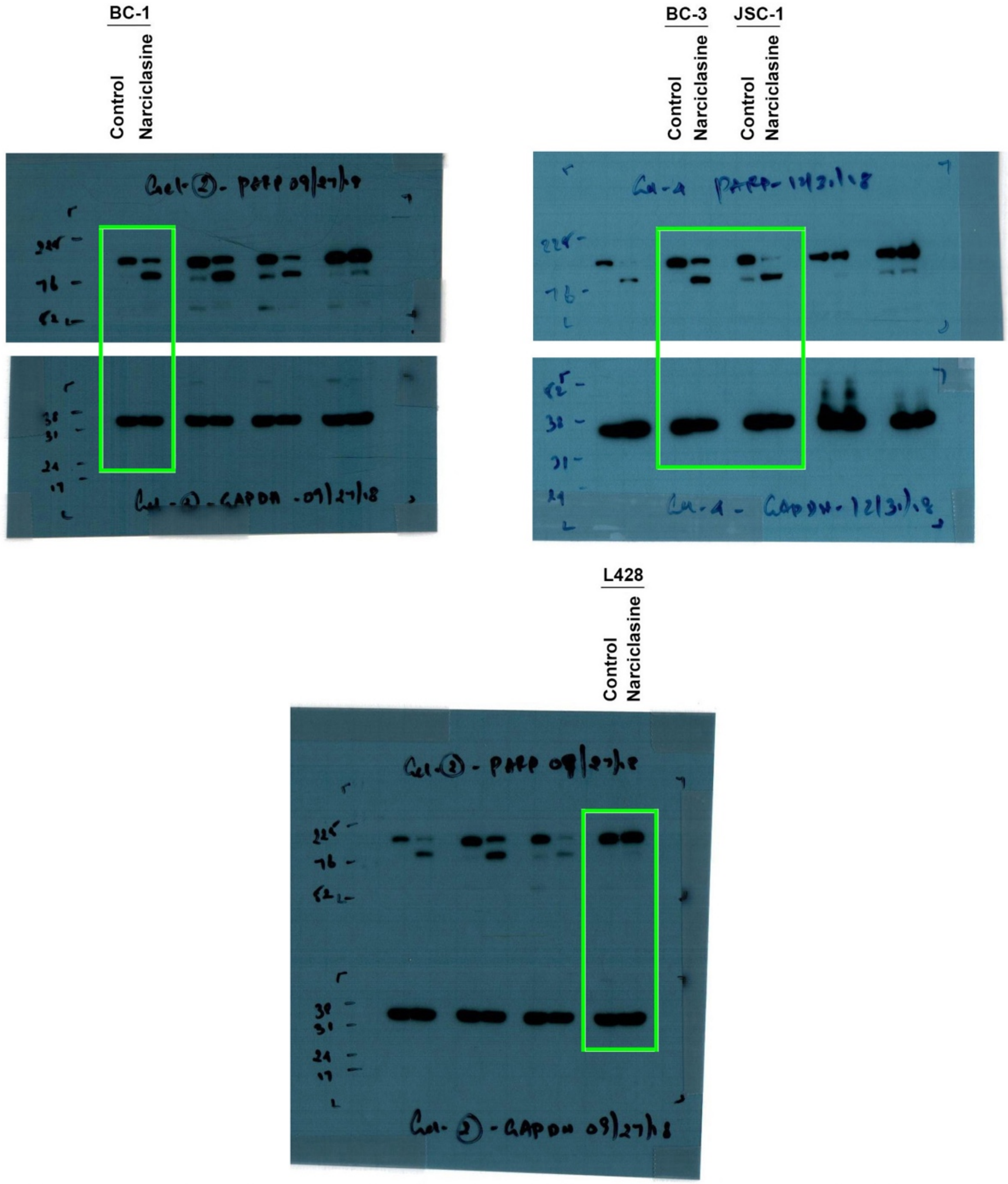

The blots were cut based on the molecular weight (MW) markers always ran on the left most lane of the gel and were incubated with specific antibodies to indicated proteins.

Supplementary figure S4: The full raw/original blots presented in figure 3C is provided here. The blots were cut based on the Molecular Weight (MW) markers. MW markers were always loaded on the left most lane of the gel. The blots were incubated with specific antibodies for indicated proteins (Details of the antibodies were given in materials and methods sections of the paper). The blots presented in figure 3C were highlighted in green rectangle area.

Supplementary figure S5: Full raw blots of figure 4A

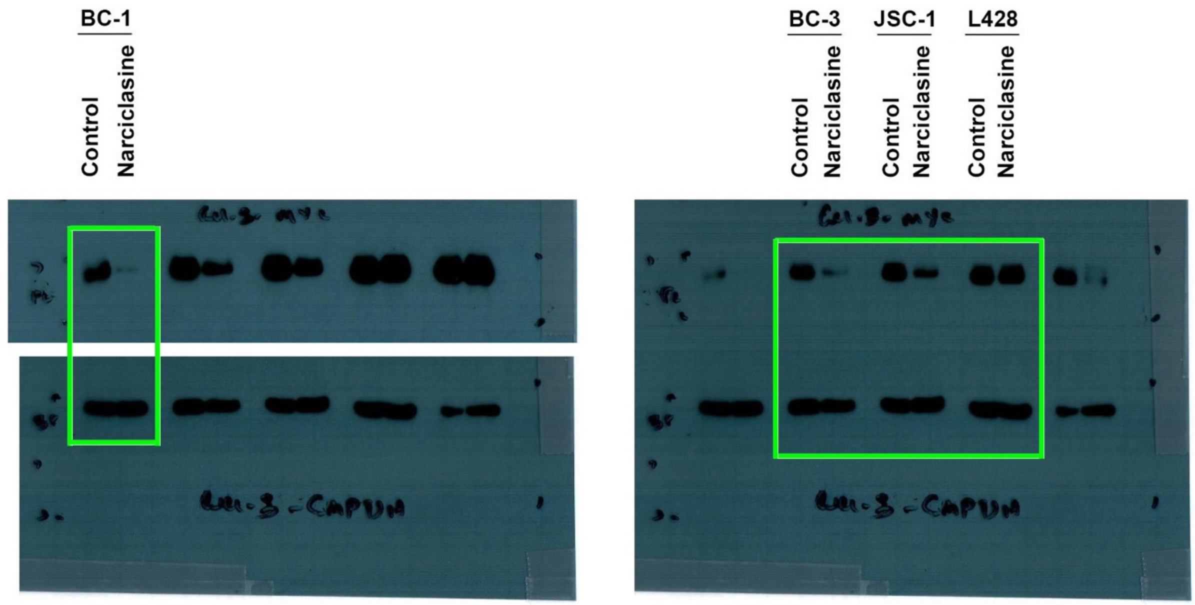

The blots were cut based on the molecular weight (MW) markers always ran on the left most lane of the gel and were incubated with specific antibodies to indicated proteins.

Supplementary figure S5: The full raw/original blots presented in figure 4A is provided here. The blots were cut based on the Molecular Weight (MW) markers. MW markers were always loaded on the left most lane of the gel. The blots were incubated with specific antibodies for indicated proteins (Details of the antibodies were given in the materials and methods sections of the paper). The blots presented in figure 4A were highlighted in green rectangle area.

Supplementary figure S6: Full raw blots of Figure 5 (PARP and its corresponding GAPDH blots)

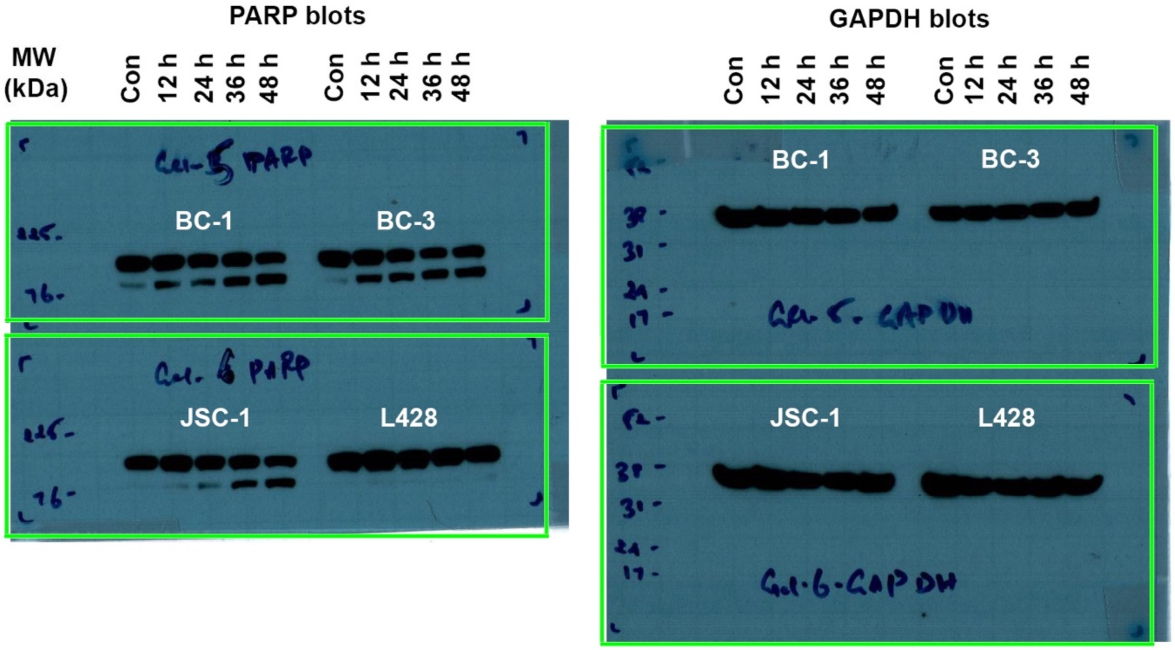

Supplementary figure S6: The full raw/original blots presented in figure 5 (PARP and its corresponding GAPDH blots) is provided here. The blots were cut based on the Molecular Weight (MW) markers. MW markers were always loaded on the left most lane of the gel. The blots were incubated with specific antibodies for indicated proteins (Details of the antibodies were given in the materials and methods sections of the paper). The blots presented in figure 5 were highlighted in green rectangle area.

Supplementary figure S7: Full raw blots of Figure 5 (MYC and its corresponding GAPDH blots)

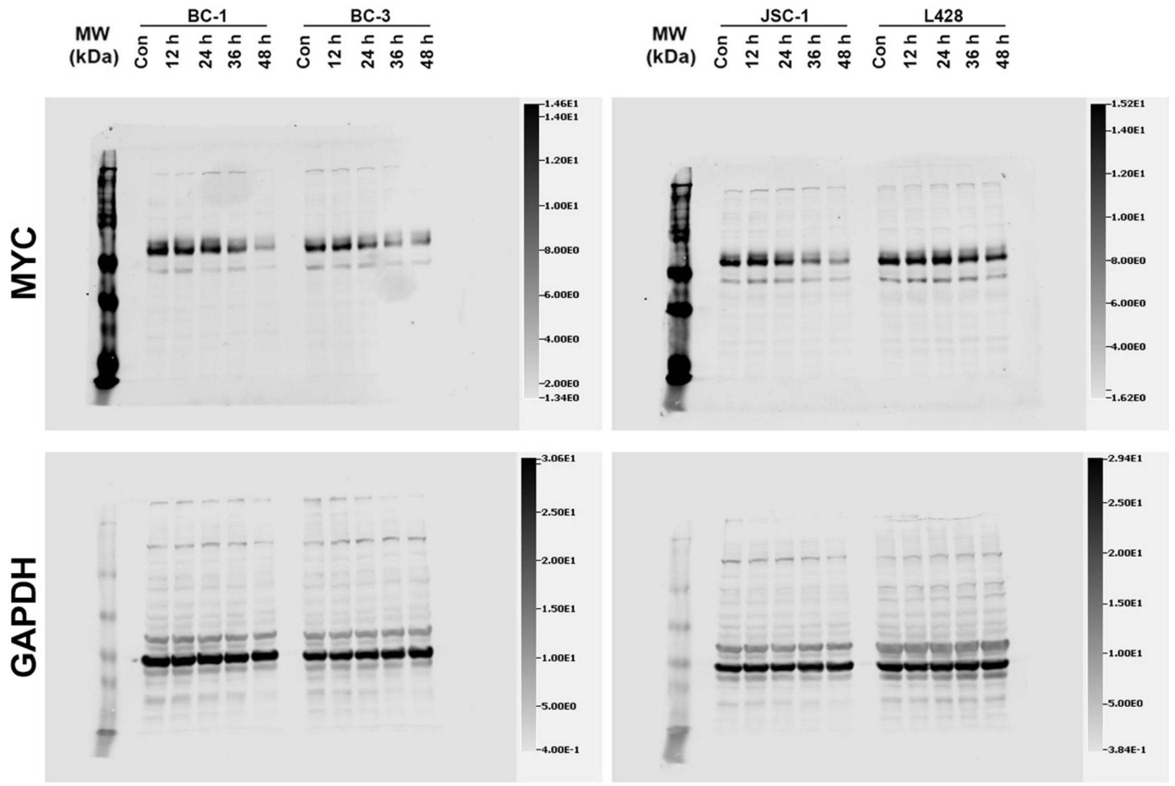

Supplementary figure S7: The full raw/original blots presented in figure 5 (MYC and its corresponding GAPDH blots) is provided here. MW markers were always loaded on the left most lane of the gel. The blots were incubated with specific antibodies for indicated proteins (Details of the antibodies were given in the materials and methods sections of the paper).

Supplementary figure S8: Full raw blots of Figure 5 (Caspase-3 and its corresponding GAPDH blots)

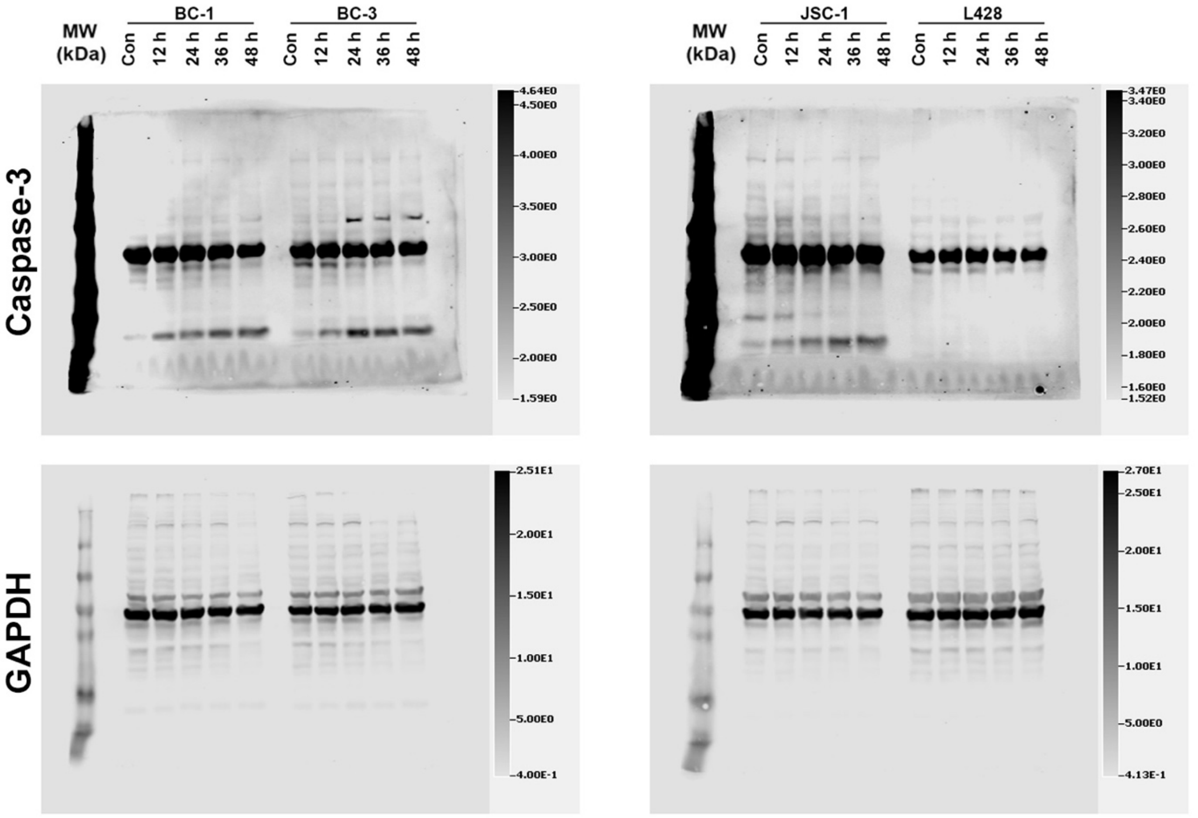

Supplementary figure S8: The full raw/original blots presented in figure 5 (Caspase-3 and its corresponding GAPDH blots) is provided here. MW markers were always loaded on the left most lane of the gel. The blots were incubated with specific antibodies for indicated proteins (Details of the antibodies were given in the materials and methods sections of the paper).

Supplementary figure S9: Full raw blots of figure 7A

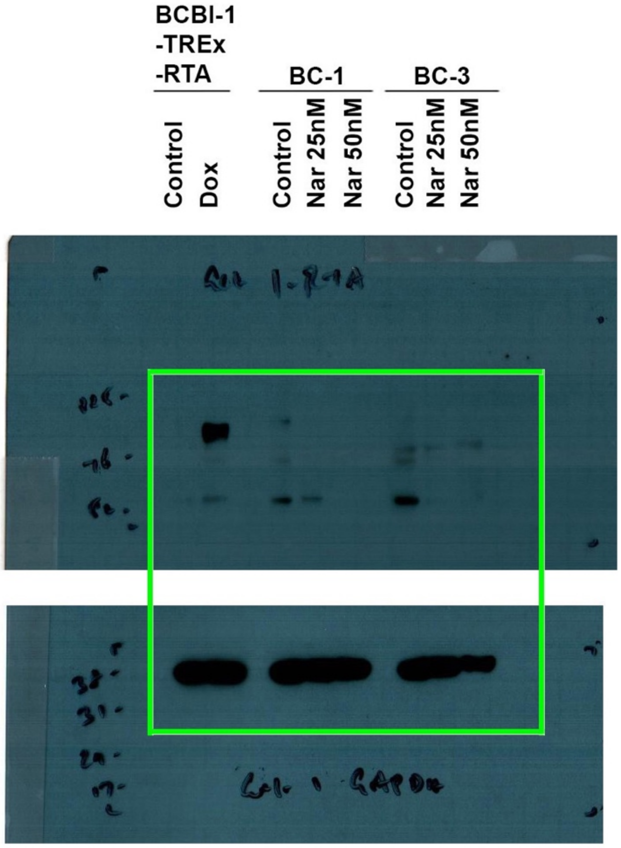

The blots were cut based on the molecular weight (MW) markers always ran on the left most lane of the gel and were incubated with specific antibodies to indicated proteins.

Supplementary figure S9: The full raw/original blots presented in figure 4A is provided here. The blots were cut based on the Molecular Weight (MW) markers. MW markers were always loaded on the left most lane of the gel. The blots were incubated with specific antibodies for indicated proteins (Details of the antibodies were given in materials and methods sections of the paper). The blots presented in figure 7A were highlighted in green rectangle area.

Supplementary figure S10: Full raw blots of supplementray figure S1A

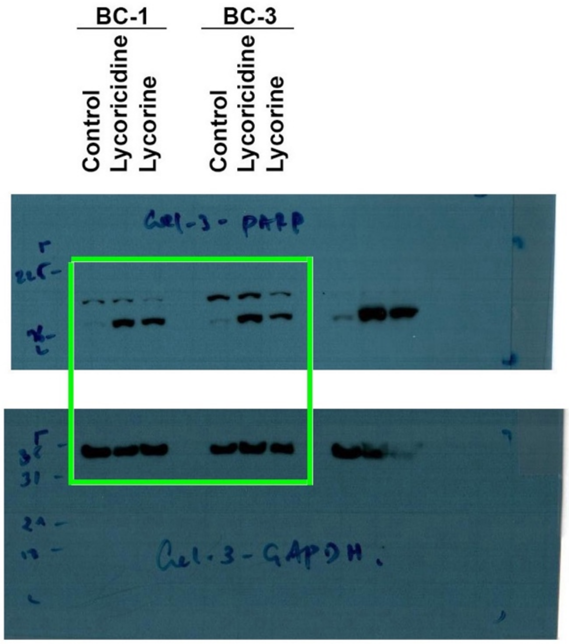

The blots were cut based on the molecular weight (MW) markers always ran on the left most lane of the gel and were incubated with specific antibodies to indicated proteins.

Supplementary figure S10: The full raw/original blots presented in supplementary figure S1A is provided here. The blots were cut based on the Molecular Weight (MW) markers. MW markers were always loaded on the left most lane of the gel. The blots were incubated with specific antibodies for indicated proteins (Details of the antibodies were given in materials and methods sections of the paper). The blots presented in supplementary figure S1A were highlighted in green rectangle area.
